# Methicillin-resistant *Staphylococcus aureus* and Vancomycin-resistant Enterococci in Rural Communities, Western United States

**DOI:** 10.3201/eid1106.050156

**Published:** 2005-06

**Authors:** Kurt B. Stevenson, Katy Searle, Gregory Stoddard, Matthew H. Samore

**Affiliations:** * Qualis Health, Boise, Idaho, USA;; † University of Utah School of Medicine, Salt Lake City, Utah, USA;; ‡ VA Salt Lake City Health Care System, Salt Lake City, Utah, USA

**Keywords:** Methicillin Resistance, Staphylococcus, Antibiotic resistance, Drug resistance, Vancomycin Resistance, Enterococci, Surveillance, Rural Communities

## Abstract

The impact and prevalence of antimicrobial drug resistance in rural community healthcare settings is uncertain. Prospective surveillance in 51 rural hospitals in Idaho and Utah examined the epidemiologic features of clinical cases of methicillin-resistant *Staphylococcus aureus* (MRSA) and vancomycin-resistant enterococci (VRE). Thirty-two cases of VRE were reported; for 6, the patient had no prior healthcare exposure or coexisting condition. Among the 724 MRSA cases available for evaluation, 405 (56%) were healthcare-associated (HA-MRSA), and 319 (44%) were community-associated (CA-MRSA). The characteristics of HA-MRSA and CA-MRSA patients with coexisting factors were similar, which suggests community transmission of healthcare strains. CA-MRSA cases without coexisting factors, however, demonstrated features previously reported for community strains. MRSA infections were substantially more frequent than VRE in rural communities in the western United States. Based on epidemiologic criteria, a large proportion of MRSA cases were community-associated. CA-MRSA rates were predictive of institutional MRSA rates.

Antimicrobial resistance is steadily rising among bacterial pathogens associated with both community- and healthcare-associated infections (1,2). Among the most important of these pathogens are vancomycin-resistant enterococci (VRE) and methicillin-resistant *Staphylococcus aureus* (MRSA) (3). Risk factors for VRE acquisition include chronic dialysis, multiple and prolonged hospitalizations, main admitting diagnosis, coexisting factors (diabetes mellitus, organ transplant, or hepatobiliary disease), previous infection or colonization with MRSA or *Clostridium difficile*, and prior treatment with antimicrobial agents (4–6). Most acquisition of VRE in the United States has occurred in the hospital or intensive care setting (4,7–9).

Risk for colonization or infection with *S. aureus* is highest in patients with diabetes mellitus, intravenous drug users, patients undergoing hemodialysis, surgical patients, and patients with AIDS (10). Additional patient risk factors for nosocomial MRSA infections, when compared to methicillin-susceptible *S. aureus*, include increased number of coexisting factors; increased length of hospital stay; exposure to antimicrobial drug agents, especially fluoroquinolones; enteral feedings; and surgery (11). In the past few years, however, reports of patients with serious MRSA infections who had no known risk factors or exposure to healthcare settings have been increasing (12–20). The distinctive properties of community-associated (CA) MRSA strains compared to nosocomial strains include a much more susceptible antimicrobial phenotype (due to the presence of a much smaller staphylococcal cassette chromosome [SCC] *mecA* [type IV]) (21) and the presence of different exotoxin gene profiles, including Panton-Valentine leukocidin (17,22,23). Patients tend to be younger and have skin and soft tissue infections or other necrotizing infections (17,21). Hospital-acquired (HA)-MRSA typically have a much larger SCC*mecA* (types I, II, and III) with a much more resistant antimicrobial phenotype (12,24).

The epidemiology of MRSA and VRE transmission may be different in the rural setting than that reported for the urban environment. For instance, inpatient acuity is substantially less in rural community hospitals than tertiary care facilities. Some of the reports of CA-MRSA infections included patients from rural communities, often native North American populations (15,16,19,23,25–28). A large study of community MRSA infection examined the characteristics of MRSA infections predominantly reported from urban and suburban regional laboratories but representing urban, suburban, and rural populations; overall, 12% of MRSA infections were community-associated by epidemiologic criteria (17).

To address the extent to which nosocomial MRSA and VRE are substantive problems in rural hospitals, we conducted a prospective study in rural Utah and Idaho. Epidemiologic and clinical data on VRE and MRSA clinical cases were collected during a 15-month period in 51 rural communities. The hospitals participating in this epidemiologic study had been surveyed about their infection control practices in 2000 (29). All had policies in place to institute contact isolation for patients with clinically recognized MRSA and VRE infection. However, none had performed active surveillance cultures to detect patients needing isolation.

## Methods

### Participating Hospitals and Study Population

Fifty-one rural hospitals in Idaho and Utah were recruited to participate in a surveillance project for antimicrobial drug resistance funded by the Centers for Disease Control and Prevention (CDC). The participating institutions represented 89% of the total number of rural hospitals in the 2 states.

All hospitals except 2 met the Office of Management and Budget definition of rural location (30). Based on this definition, a rural county was considered any county that did not have a metropolitan center with a population exceeding 50,000 persons. Hospitals within such counties were considered to meet this rural definition. Among the 2 participating hospitals not meeting this definition, 1 hospital was in an isolated county slightly exceeding the 50,000 metropolitan population limit but was considered to serve a primarily rural population. The second was a small hospital (50 beds) located within but at the border of an urban county.

### Surveillance System

Clinical cases of VRE and MRSA identified by the clinical laboratories of participating hospitals were reported to the respective infection control practitioners, who compiled demographic, medical history, and other epidemiologically relevant data on each case. Individual level race and ethnicity were not captured.

In some cases, the microbiology staff contributed to data collection. The primary source of information was the patient's medical record. In most cases direct confirmation of healthcare exposure through patient or family interview was not possible. These data were recorded on a standardized data collection form and submitted on a regular, usually monthly, schedule. Data evaluated in this analysis were collected from October 1, 2002, to December 31, 2003. All data collected and analyzed were for this 15-month period, except for incidence rate calculations, which were for calendar year 2003, as described below. The following approaches were used to improve the validity of the data: 1) a data dictionary and operations manual were created with explicit instructions for completion of the data collection forms; 2) the data collection protocol was discussed during conference calls along with frequent one-on-one communication; and 3) anomalous data in the data reports were routinely searched for and corrected.

### Epidemiologic Definitions

Definitions used in this study focused on the location of the patient at the time of initial culture and the presence or absence of exposure to the healthcare environment. The emphasis, therefore, is on healthcare or community association rather than definitive identification of the site of acquisition. These definitions are consistent with those of CDC and others (16–18,20).

### Healthcare-associated VRE (HA-VRE) or MRSA (HA-MRSA)

This category included VRE or MRSA cultured from patients >48 hours after hospital admission, or while a patient of another hospital, resident of a long-term care facility, or transitional care unit. Also included in this category were infected patients with history of prior hospitalization or outpatient surgery, prior residence in a long-term care facility or transitional care unit, or prior care from home health agency or with documented indwelling catheters. This group also included patients with a postoperative wound infection, even if the surgery was performed as an outpatient. Patients identified with any of the above healthcare exposures were included in this category; the period from healthcare exposure to inclusion was generally 6 months. Patients known to have previous VRE or MRSA infections or positive cultures were excluded from analysis.

### Community-associated VRE (CA-VRE) or MRSA (CA-MRSA)

This category included VRE or MRSA cultured from patient <48 hours after hospital admission, or as an outpatient. Excluded from this category were patients with previous history of positive VRE or MRSA culture or infection, prior hospitalization or outpatient surgery, prior residence in a long-term care facility or transitional care unit, or prior home health.

### Coexisting Factors

This category included medical or other factors possibly associated with healthcare exposure (diabetes mellitus, renal failure, prior antimicrobial drug therapy, and immunosuppression). Such factors were assessed for all groups of VRE and MRSA cases.

### CA-VRE or CA-MRSA with Coexisting Factors

This category included VRE or MRSA clinical cases satisfying the definition for community-associated infection in which the patient had identifiable coexisting factors.

### CA-VRE or CA-MRSA without Coexisting Factors

This category included VRE or MRSA case satisfying the definition for community-associated infection in which the patient had no identifiable coexisting factors.

### Incidence Rates

The incidence of CA- and HA-VRE and MRSA were calculated for each hospital reporting positive cultures from January 1, 2003, to December 31, 2003. Individual institutions were excluded from this analysis if the respective hospital infection control and microbiology staff could not verbally attest to the completeness of reporting after the study ended, based on a retrospective review of all cases during the study period. Thus, hospitals with partial or incomplete reporting of all cases were not included in the incidence rate calculations or Poisson regression model. The described hospital located at the border of the urban county was also excluded from the incident rate calculations. The denominator for healthcare-associated cases was the total inpatient census for 2003, including long-term care facility census if a hospital had such an attached facility. These data were obtained from published hospital statistics (31) or by direct communication with hospital staff. The denominator for community-associated infections was the most recent estimated county population data derived from the 2000 census in Idaho (Idaho Department of Health, http://www2.state.id.us/dhw/vital_stats/health_district_report.pdf) and Utah (Utah Department of Health, http://health.utah.gov/vitalrecords/pub_vs/ia02/02bx.pdf). In both cases, the incidence rates were expressed as the number of hospital cases per 10,000 patient-days or number of community cases per 10,000 person-years, based on county population. Incidence rates for CA-MRSA were also calculated by using the community population size as the denominator. For comparison, MRSA incidence rates for CDC National Nosocomial Infections Surveillance (NNIS) hospitals during 2003 were obtained (T. Horan and J. Edwards, pers. comm.).

### Susceptibility Testing

Antimicrobial susceptibility results, collected retrospectively, were not available for all MRSA cases. Available data were aggregated and compared among the different groups of MRSA patients. Specifically, clinical and epidemiologic characteristics of MRSA cases for which the infecting organisms were resistant to both clindamycin and ciprofloxacin ("resistant group") and MRSA cases for which the infecting organisms were sensitive to both clindamycin and ciprofloxacin ("susceptible group") were compared. Phenotypic susceptibility to these 2 antimicrobial agents in MRSA is most often associated with community acquisition (17,24).

## Data Analysis

All data were entered into an Access (Microsoft Corporation, Redmond, WA, USA) relational database for analysis. Proportions of total cases meeting specific epidemiologic criteria were calculated, and characteristics of each category were compared by using Fisher exact testing. Hospital bed size, census data, and age for MRSA patients were available for most but not all entries. Census data and ages of patients in each category were compared with Kruskal-Wallis equality of populations rank test. The relationship of community MRSA rates and other covariates on the hospital MRSA rates were modeled by using random effects Poisson regression. In this model, each specific hospital was considered a unit and was treated as a random effect; its MRSA cases were assumed to be correlated. That is, the hospitals were considered to be a random sample of all rural Utah and Idaho hospitals. This assumption permitted inferences to be made to this target population, rather than limiting inferences to only those hospitals included in the model. Continuous predictor variables were converted to ordered categoric variables and included in the models as tertiles, quartiles, or quintiles to verify that risk was "linearly" increasing. Multivariable models were fitted by using backwards stepwise variable selection. All statistical testing was performed with STATA, version 8 (Stata Corporation, College Station, TX, USA). All statistical analyses were 2-sided and significance was set at p<0.05.

## Results

### Case Ascertainment

A total of 34 unique VRE and 799 unique MRSA cases were reported by participating rural healthcare institutions in Idaho and Utah from October 1, 2002, to December 31, 2003. Twenty-six of 51 institutions reported ≥1 MRSA or VRE case during this interval. Infection control practitioners or microbiology staff from 28 institutions confirmed that reporting was complete; 9 of the 28 institutions with confirmed complete reporting had no MRSA cases, and 23 of the 28 institutions had no VRE cases during the interval. The 22 institutions that did not attest to complete reporting contributed 17 MRSA and 2 VRE cases to the descriptive case series analysis but were removed from the incidence rate analysis. The average bed size of institutions with complete reporting was greater than the average bed size of institutions that did not attest to complete reporting (53 beds vs. 29 beds, p = 0.03).

Two VRE (6%) and 75 MRSA (8%) cases were excluded from the case-series analysis because of incomplete data. Of the 32 VRE cases with complete data, criteria for HA-VRE infection and CA-VRE infection were met by 25 (78%) and 7 (22%), respectively. Of the remaining 724 MRSA cases, 405 (56%) were HA-MRSA infection and 44% were CA-MRSA infection. Among the CA-MRSA cases, 79 (25%) were from patients with known coexisting factors, and 240 (75%) came from patients without any such factors reported (Table 1).

**Table 1:** Characteristics of VRE and MRSA clinical cases*

Characterization	VRE		MRSA	
	No. (N = 32)	%	No. (N = 724)	%
Healthcare-associated	25	78	405	56
Community-associated	7	22	319	44
without coexisting factors	6	19	240	33
with coexisting factors	1	3	79	11
Location of time of culture				
Community	9†	28	391‡	54
Ward	4	13	113	16
Intensive care unit	0	0	24	3
Long-term care facility	6	19	147	20
Transitional care unit	12	38	19	3
Other hospital	1	3	29	4
Clinical sources				
Skin and soft tissue	4	13	400	55
Urine	15	47	104	14
Blood	2	6	38	5
Sputum	0	0	116	16
Other	11	34	64	9
Unknown	0	0	2	1
Coexisting factors				
>65 years of age	19	59	353	49
Diabetes mellitus	4	13	142	20
Renal failure	3	9	61	8
Prior antimicrobial therapy	5	16	210	29
Immunosuppression	6	19	77	11
Sex				
Male	14	44	382	53
Female	18	56	342	47

*VRE, vancomycin-resistant enterococci; MRSA, methicillin-resistant *Staphylococcus aureus*. †There were 2 VRE cases in which cultures were obtained in the community setting in patients with previous history of healthcare exposure (recent hospitalization, residence in long-term care facility). ‡There were 72 MRSA cases in which cultures were obtained in the community setting in patients with previous history of healthcare exposure. The location at the time of culture was unknown in one MRSA case.

### Characteristics of VRE and MRSA Clinical Cases

Eight institutions reported at least 1 VRE case. The major healthcare location of patients with HA-VRE was the transitional care unit (12/32, 38%); other locations are outlined in Table 1. Six patients (19%) had no reported risk factors or known exposure to the healthcare setting. The most common clinical source of VRE isolates was urine (15/32, 47%). The major location for patients at the time of MRSA culture was the community (391/724, 54%) with the long-term care facility (147/724, 20%) representing the most common healthcare location (Table 1). The most common clinical source of MRSA cultures was skin and soft tissue (400/724, 55%).

The clinical sites of infection for all MRSA cases were compared (Table 2). Comparison of clinical sources for MRSA infection between groups of HA-MRSA and CA-MRSA patients with coexisting factors showed no significant differences (data not shown). Patients in the CA-MRSA group without coexisting factors, however, had the highest proportion of skin/soft tissue infections (156/240, 65%, p<0.0001). Patients in this group were also much younger (mean age 41.5 years, n = 178) than the other 2 groups (mean ages 68.8, n = 357 and 59.0 years, n = 66) (p = 0.0001); the proportion of patients <20 years of age in this group was 24% (43/178) compared to 2% (6/351) and 8% (5/66) in the other 2 groups (p<0.0001). Among hospitals with complete reporting, the fraction of MRSA cases that were CA-MRSA (with and without coexisting factors) was slightly higher in smaller hospitals compared to larger hospitals, but this difference was not significant (48% if bed size <40 and 41% if bed size >40, p = 0.323).

**Table 2:** Clinical sources for MRSA cultures*

Clinical source	No. (%)			
	HA-MRSA, N = 405	CA-MRSA with CFs, N = 79	CA-MRSA without CFs, N = 240	p value†
Skin and soft tissue	197 (49)	47 (60)	156 (65)	<0.0001
Urine	61 (15)	11 (14)	32 (13)	NS
Blood	33 (8)	2 (3)	3 (1)	<0.0001
Sputum	89 (22)	12 (15)	15 (6)	<0.0001
Other	23 (6)	7 (9)	34 (14)	0.001
Unknown	2 (1)	0 (0)	0 (0)	NS

*MRSA, methicillin-resistant *Staphylococcus aureus*; HA, heathcare-associated; CA, community-associated; CFs, coexisting factors; NS, not significant. †Based on Fisher exact test.

Susceptibility to antimicrobial agents was compared among these 3 epidemiologic groups of MRSA infections (Table 3). Susceptibility to 3 key non-β-lactam antimicrobial agents (erythromycin, clindamycin, and ciprofloxacin) was significantly higher in the group of CA-MRSA patients without coexisting factors than in the other 2 groups. No statistical difference in the susceptibility to erythromycin, clindamycin, or ciprofloxacin existed between HA-MRSA and CA-MRSA groups with coexisting factors (data not shown).

**Table 3:** Comparison of antimicrobial susceptibilities by category

Agent	No. susceptible (%)			p value†
	HA-MRSA	CA-MRSA with CFs	CA-MRSA without CFs	
Oxacillin	0/204 (0)	0/34 (0)	0/106 (0)	NS
Erythromycin	10/193 (5)	3/34 (9)	16/98 (16)	0.007
Clindamycin	42/195 (22)	11/34 (32)	61/100 (61)	<0.0001
Ciprofloxacin	9/138 (7)	2/19 (11)	23/73 (32)	<0.0001
Gentamicin	196/204 (96)	33/34 (97)	99/105 (94)	NS
Trimethoprim-sulfamethoxazole	202/203 (99)	34/34 (100)	102/106 (96)	NS
Rifampin	157/166 (95)	23/24 (96)	79/82 (96)	NS
Tetracycline	183/190 (96)	25/27 (93)	93/101 (92)	NS
Vancomycin	203/203 (100)	33/33 (100)	105/106 (99)‡	NS

*HA, healthcare-associated; MRSA, methicillin-resistant *Staphylococcus aureus*; CA, community-associated; CFs, coexisting factors; NS, not significant. †Based on Fisher exact test. ‡One isolate reported as vancomycin resistant. Isolate not available for confirmatory testing.

MRSA cases in which the isolate was resistant to both clindamycin and ciprofloxacin (n = 142) were compared to cases in which both antimicrobial agents were susceptible (n = 32) (Table 4). The proportion of patients with skin/soft tissue infections in the susceptible group (81% vs. 52%, p = 0.003) increased significantly. Most of the cases in the susceptible group were community-associated (75% vs. 27%, p<0.0001), and the mean age of the susceptible group was significantly lower (32 vs. 69, p = 0.0001).

**Table 4:** Comparison of MRSA cases with resistant and susceptible phenotypes*

	No (%)		
	Resistant group†, N = 142	Susceptible group‡, N = 32	p value§
Skin and soft tissue	74 (52)	26 (81)	0.003
Urine	15 (11)	0 (0)	NS
Blood	9 (6)	1 (3)	NS
Sputum	30 (21)	1 (3)	0.019
Other source	14 (10)	4 (13)	NS
Community-associated	38 (27)	24 (75)	<0.0001
Sex			
Male	79 (56)	17 (53)	–
Female	63 (44)	15 (47)	–
Mean age (y)	69	32	0.0001
Age >65 y	89 (63)	3 (9)	<0.0001
Diabetes mellitus	38 (27)	1 (3)	0.002
Renal failure	13 (9)	0 (0)	NS
Prior antimicrobial therapy	61 (43)	6 (19)	0.015
Immunosuppression	14 (10)	0 (0)	NS

*MRSA, methicillin-resistant *Staphylococcus aureus*; NS, not significant. †MRSA isolates resistant to both clindamycin and ciprofloxacin. ‡MRSA isolates susceptible to both clindamycin and ciprofloxacin. §Based on Fisher exact test. Comparison of age tested by Kruskal-Wallis equality of populations rank test.

### Comparison of MRSA and VRE Incidence Rates

Incidence rates of VRE and MRSA infections, particularly of HA-MRSA, varied substantially across institutions (Figure). Rates of CA-MRSA correlated strongly with HA-MRSA rates, regardless of whether CA-MRSA rates were denominated by community or county population size (Table 5). The rate of HA-MRSA in hospitals belonging to the third quintile of CA-MRSA rates was 11-fold higher than in hospitals with no CA-MRSA (first and second quintiles). The rate of HA-MRSA in hospitals belonging to the fourth and fifth quintiles of CA-MRSA rates was >30-fold higher than in hospitals with no CA-MRSA. This association was independent of hospital bed size.

**Figure:**
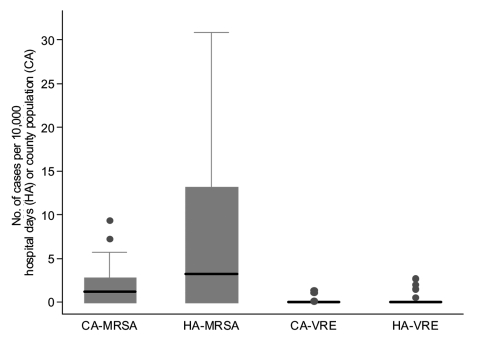
Box plot of incidence rates of methicillin-resistant *Staphylococcus aureus* (MRSA) and vancomycin-resistant enterococcal (VRE) infections. CA, community-associated; HA, healthcare-associated.

**Table 5:** Random effects Poisson regression model for HA-MRSA* rate†

Predictors	No. of institutions	Incidence rate ratio	95% CI	p value
Quintile of CA-MRSA rate‡				
1st–2nd: 0	11	Reference		
3rd: 0.3 to 1.6	6	11	2.8–4.5	0.001
4th: 1.7 to 3.4	6	35	10–122	<0.0001
5th: 3.5 to 9.4	5	33	9.2–115	<0.0001
Hospital bed size				
13–25	10	Reference		
26–50	9	0.8	0.3–2.0	0.596
51–235	9	1.3	0.6–3.0	0.530
State				
Idaho	17	Reference		
Utah	11	2.3	1.3–4.2	0.005

*HA-MRSA, heathcare-associated methicillin resistant *Staphylococcus aureus*; 95% CI, 95% confidence interval; CA-MRSA, community-associated MRSA. †No. of HA-MRSA cases/10,000 occupied bed-days. ‡No. of CA-MRSA cases/10,000 person-years, based on county population.

MRSA incidence was also examined in relation to proximity to Native American reservations. HA-MRSA and CA-MRSA incidence rates at the 7 communities that were in proximity to Native American reservations were comparable to other communities. Only 1 of these 7 had CA-MRSA rates in the highest quintile.

## Discussion

Much of our current understanding of MRSA and VRE in rural communities comes from reports of outbreaks or smaller case series (15,16,19,23,25–28). In this study, epidemiologic data on MRSA and VRE cases were collected from a large number of rural hospitals in Idaho and Utah during a 15-month period. VRE incidence was low in all but 3 institutions. The overall incidence of MRSA infection was substantially greater and varied widely across different institutions. Rates of HA-MRSA were significantly higher in communities classified as having a high incidence of CA-MRSA. The incidence rates of HA-MRSA in communities that did report cases were in a range comparable to reports from hospitals participating in the CDC NNIS system (HA-MRSA incidence rates from NNIS hospitals ranged from 12.6 to 19.5 per 10,000 patient days) (T. Horan and J. Edwards, pers. comm.). The lack of reporting of MRSA and VRE from many rural hospitals suggests that these resistance types may still be infrequent in some locations. However, confirming this finding by performing active surveillance cultures to determine whether the prevalence of carriage is correspondingly rare in communities without clinical cases would be useful.

Forty-four percent of the MRSA cases met epidemiologic criteria for being community-associated. These cases were comparable to CA-MRSA cases reported by other investigators with respect to the infrequency of coexisting factors, the predominance of skin and soft tissue infection, and the increased susceptibility to other antimicrobial drug classes (12–18). Some cases in the CA-MRSA group without coexisting factors had a more resistant phenotype, which suggests that, even in these patients without obvious risk factors, healthcare exposure to MRSA occurred, or descendants of hospital strains of MRSA were available in the community for transmission. The subset of CA-MRSA cases with coexisting factors had characteristics similar to cases meeting the epidemiologic criteria for healthcare acquisition. Transmission of healthcare-associated strains in these cases may have occurred during contact in ambulatory rather than hospital settings. These hypotheses are supported by the work of other investigators (18,32–34). In 1 study, molecular analysis of CA-MRSA isolates in a nonoutbreak setting demonstrated that the hospital was the main source of community MRSA (33).

We found that the rate of hospital-associated MRSA was significantly greater in communities classified as having a high incidence of CA-MRSA. Several plausible reasons for this association exist. One possibility is that the sensitivity of laboratory detection and reporting was better in communities that had increased rates of both CA- and HA-MRSA. Another potential explanation is that CA- and HA-MRSA cases are dynamically interdependent (35). CA-MRSA cases may contribute to nosocomial dissemination of MRSA within hospitals because of increased prevalence of MRSA carriage at the time of admission, followed by transmission to other hospitalized patients (36,37). Increased HA-MRSA incidence may in turn foster dissemination of MRSA in community populations.

A smaller but substantial proportion of the VRE cases (19%) also met criteria for community association, without other risk factors. VRE transmission from farm animals to humans has been reported in Europe, and community transmission has been suggested as a possible but yet undocumented mechanism in the United States (7,9).

This study has several limitations. Clinical microbiology laboratories, particularly those in rural hospitals, may have difficulty detecting MRSA, VRE, and other resistant organisms (38–40). We did not directly evaluate proficiency testing in the current study but have examined this issue in prior investigations. Laboratory practices in rural hospitals in Idaho and Utah were examined by survey in July 2000 (40). Five institutions in the current study that had inadequate MRSA confirmation procedures according to the survey conducted in 2000 had rates of HA-MRSA that were comparable to institutions with adequate procedures for confirmation of MRSA. In a follow-up study, laboratory proficiency was assessed by distribution of unknown specimens for blinded testing to a subset of 29 facilities in Idaho and Utah (K.B. Stevenson et al., unpub. data). Reporting of interpretative category for MRSA and VRE was correct in 100% and 57% of hospitals, respectively. These results highlight the potential for problems in microbiology proficiency to contribute to either underdetection or overdetection of resistant organisms.

Another potential drawback of this study was that original records such as hospital charts were not independently reviewed to assess the reliability of epidemiologic data collection by local infection control practitioners. Our use of an explicit data collection protocol and data dictionary was designed to mitigate this limitation. Similar methods of medical record review have been used successfully in other studies of CA-MRSA (15,16).

This study focused on patients with clinical infection. Because serial surveillance cultures were not obtained, the timing or location of VRE or MRSA acquisition could not be precisely determined. Surveillance cultures also identify patients with clinically unrecognized carriage of resistant organisms. MRSA isolates were not collected prospectively during the study period, which limited our ability to confirm antimicrobial drug–susceptibility patterns and perform molecular analysis. Examining the clonal patterns of isolates by pulsed-field gel electrophoresis and determining the type of SCC*mecA* would have been useful for supporting the interpretations of the epidemiologic analysis and overcoming the recognized limitations. Molecular analyses of MRSA isolates that have been recently collected from these rural communities are planned.

Finally, the number of persons corresponding to the source population for CA-MRSA and CA-VRE cases could not be precisely determined. Therefore, we could not derive reliable estimates of CA-MRSA and CA-VRE infection rates for purposes of comparison with rates from other geographic locations.

In summary, infection control practitioners and clinicians working in rural areas are likely to confront problems of hospital- and community-associated MRSA infection. In some rural areas, the MRSA incidence approaches or exceeds what has been reported from larger hospitals in urban areas. The role of more aggressive prevention strategies, such as active surveillance culturing, in these rural healthcare settings is still uncertain. Further studies of the epidemiologic factors that influence MRSA and VRE transmission and of infection control interventions in rural communities are needed.

Funding was provided by CDC, grant number RS1 CCR820631.
